# Validation of the Georgian Version of the Multiple Sclerosis Quality of Life-54 (MSQOL-54) Questionnaire for Assessing the Quality of Life in Patients With Multiple Sclerosis

**DOI:** 10.7759/cureus.73881

**Published:** 2024-11-17

**Authors:** Natalia Khutsishvili, Elza Nikoleishvili, Tina Beruchashvili, Otar Toidze, Marika Megrelishvili, Nino Ganugrava

**Affiliations:** 1 School of Health Sciences and Public Health, University of Georgia, Tbilisi, GEO; 2 School of Medicine, Ilia State University, Tbilisi, GEO; 3 School of Medicine, University of Georgia, Tbilisi, GEO

**Keywords:** expanded disability status scale, georgia, health-related qol, msqol-54, multiple sclerosis

## Abstract

Background and objectives: Multiple sclerosis (MS) significantly impacts health-related quality of life (HRQOL). The Multiple Sclerosis Quality of Life-54 (MSQOL-54) is a widely used tool for assessing HRQOL in individuals with MS. Adapting this tool to the local language and culture is essential for ensuring cultural relevance, enabling comprehensive care, and allowing international comparability. This study aims to validate the Georgian version of the MSQOL-54 (MSQOL-54 GEO) and assess HRQOL in MS patients in Georgia.

Methodology: A cross-sectional study was conducted, involving 384 individuals with MS. The MSQOL-54 questionnaire was translated using a forward-backward translation technique. Statistical analysis, performed using SPSS 22.0, included internal consistency reliability measured by Cronbach's alpha coefficient (α) with 95% confidence intervals (95% CI), while external validity was determined through the correlation of individual components and composite scores with the Expanded Disability Status Scale (EDSS).

Results: The MSQOL-54-GEO demonstrated strong validity and reliability, with most components showing Cronbach's alpha values above 0.70, except for the "Social Function" component. The "Health Perceptions" component recorded the lowest average score (47.3 ± 20.0). Significant correlations were found between MS duration and "Physical Health," as well as "Cognitive Function."

Conclusions and implications: The findings indicate that the MSQOL-54-GEO is a reliable, convenient, and accepted tool for assessing HRQOL among MS patients in Georgia. This validation contributes valuable insights into the internal consistency of the MSQOL-54-GEO, facilitating its essential use in clinical practice and research, for evaluating treatment outcomes and guiding holistic care approaches, encompassing physical, emotional, and social well-being.

## Introduction

Multiple sclerosis (MS) is a chronic, inflammatory disorder that specifically affects myelin in the central nervous system (CNS), resulting in a wide range of neurological symptoms and impairments [1]. Around 2.8 million people worldwide have been diagnosed with MS, and the number of cases has consistently increased since 2013. Researchers show a notable gender disparity, with the female-to-male prevalence ratio ranging from approximately 2:1 to 3:1, depending on the region, highlighting a higher prevalence among women [2]. MS is a leading factor causing neurological impairment among young adults. MS commonly affects younger age group (20-40) and has a significant impact on individuals, families, and healthcare systems [3].

The health-related quality of life (HRQOL) of patients with MS includes physical, psychological, and social factors. Individuals with MS often experience a decreased HRQOL as the disease progresses. The social consequences of MS, such as poor social attitude, difficulties in finding work, and limited engagement in social activities, increase the negative effects on HRQOL. Understanding the connection between HRQOL and social burden is critical for developing comprehensive interventions that meet the needs of individuals with MS and improve their overall well-being [4].

The MSQOL-54 questionnaire, originally developed in the United States in 1995, is a key instrument for assessing the HRQOL in individuals with MS. The assessment performs an in-depth evaluation, ensuring that it is valid, reliable, culturally and linguistically adaptable, and focused on patient-centered outcomes. The MSQOL-54 evaluates various aspects of HRQOL, such as physical and mental well-being, limitations in daily activities caused by physical and emotional factors, pain, lack of energy levels, emotional state, social functioning, cognitive abilities, health-related distress, sexual function, satisfaction with sexual function, overall quality of life, and changes in health status over time [5]. The adaptation of the MSQOL-54 questionnaire to the Georgian context ensures that it more accurately reflects the lived experiences of Georgian MS patients. Cultural adaptation helps address language, social expectations, and healthcare-seeking behaviors, which are essential for creating valid and reliable measures of QoL in specific cultural settings and diverse populations. Assessing the QOL of MS patients using internationally recognized tools like the MSQOL-54, translated and validated in the local language, is crucial for several reasons: 1) cultural relevance (it ensures that the tool captures the specific nuances of the local culture, health beliefs, and language, leading to more accurate results); 2) comprehensive care (understanding patients' QOL allows healthcare providers to address both physical and psychosocial needs, creating a more holistic care approach); 3) international comparability (using a standardized tool, even in translation, enables comparison with international studies, which can highlight global trends, differences, and similarities, helping to tailor interventions more effectively). MS causes specific social and economic obstacles, including discrimination, work boundaries, financial strain, and social isolation. Because of the complicated relationship between MS symptoms, functional limitations, and psychological concerns, it is critical to comprehensively investigate and assess patients' HRQOL [6]. This article aims to emphasize the challenges experienced by MS patients and advocate for comprehensive measures to improve their well-being and general QoL by examining the various aspects of HRQOL in MS.

The study is the initial effort to examine HRQOL in MS patients in Georgia. The primary objective is to validate the Georgian version of the MSQOL-54, which will be used to assess the QoL of MS patients using a standardized tool.

## Materials and methods

Methodology

A cross-sectional study was conducted at six primary service provider MS centers, in Georgia (S. Khechinashvili University Clinic, Institute of Neurology, Pineo, Mediclub, Aversi, and Curacio). The research was conducted in compliance with bioethical standards, following the approved research protocol, and obtaining a license from the UG Ethical Commission (Institutional Review Board, UGREC-04-23), with the aim of ensuring the patients' confidentiality. The study was scheduled for execution from May to December of 2023. The sample size was determined using a statistical technique.

n = z^2^*p̂(1-p̂)/e^2^, ​ 

z = 1.96; (p ) ^= 0.5 (since there are no official data on the prevalence of MS in Georgia, the proportion was approximated to be 50%); ε = 0.05 (margin of error - 5%).

The study included 384 people diagnosed with MS. The selection method included criteria for inclusion (age > 18 years old, MS diagnosis, and completion and signing of a paper-based informed consent form) and exclusion (refusal to participate or psychiatric illnesses).

Translation and adaptation of the MSQOL-54 questionnaire

The MSQOL-54 questionnaire was translated and adapted using the forward-backward translation method, as defined by Beaton and co-authors [7, 8]. Two different translators worked on the translation, which was further reviewed by two multilingual neurologists (not affiliated with the clinics, involved in the research). Two translators, working independently, translated the questionnaire from English to Georgian. Following that, a panel of bilingual specialists did a comprehensive assessment of the translation. After that, two translators independently translated the questionnaire from Georgian to English, which was then reviewed by a second team of experts (two independent neurologists). Finally, the panel of professionals assessed the translation's adequacy and made the necessary changes to ensure it was consistent with Georgia's cultural environment.

The preliminary investigation included a cohort of 30 MS patients who spoke two languages (English and Georgian). Participants' English proficiency was assessed informally, through a volunteer-based approach. The patients, who felt comfortable with their understanding of English participated, ensuring that responses accurately reflected the individuals' experiences without the need for interpreter support. The participants were given the original version of the questionnaire to complete. After seven days, the participants received the translation of the questionnaire into Georgian. In the pilot study phase, feedback from participants was instrumental in assessing the clarity and grammatical accuracy of the translated MSQOL-54 items. This feedback allowed the researchers, to make necessary adjustments to ensure each item was easily understood, culturally relevant, and linguistically accurate for Georgian MS patients.

Statistical methods

The statistical analysis was performed by using Statistical Package for the Social Sciences (SPSS) 22.0 software (IBM Corp., Armonk, NY). Continuous variables are represented by their means, standard deviations (SD), medians, and minimum-maximum ranges. The Cronbach α coefficient was used to assess internal consistency reliability, with a range of 0 to 1, with 95% confidence intervals (95%CI).

Internal validity was assessed using Pearson's R correlation coefficients. The correlation coefficients between each domain and its corresponding composite score were compared to those of the other domains and respective composite scores. A value of R greater than 0.100 was considered statistically significant, with a p-value of less than 0.05.

External validity was evaluated by calculating the correlation between each individual component and composite scores to the EDSS score. The scores for each dimension and domain of MSQOL-54-GEO and EDSS were expected to be negatively correlated. The Pearson correlation coefficients were used to assess the association between composite scores and a variety of clinical and demographic factors, including age, age at MS diagnosis, MS duration, place of residence, education, occupation, family status, type of treatment, and disability status.

## Results

The main findings of this study revealed that the Georgian version of the MSQOL-54 questionnaire demonstrated good internal consistency and reliability, with Cronbach’s alpha coefficients exceeding the accepted thresholds for each subscale. In addition, the data showed that the quality of life among MS patients in Georgia was significantly impacted by physical health limitations, fatigue, and emotional well-being, aligning with findings from international studies. These results provide strong evidence for the cultural validity of the questionnaire and underscore the importance of contextually relevant assessments in understanding the quality of life in MS patients.

The research study included a cohort of 384 MS patients, as shown in Table 1. There were 268 women in the study population, representing 69.8%. The predominant age groups were those aged 30 to 39 (35.4% of the population) and those aged 40 to 49 (32.6% of the population). The majority of patients (60.9%) lived in urban areas of Georgia.

**Table 1:** Characteristics of the Georgian MS patients (n = 384) RRMS: relapsing-remitting multiple sclerosis, EDSS: Expanded Disability Status Scale, MS: multiple sclerosis

Characteristics	n (%)		
Gender			
Male	116 (30.2%)		
Female	268 (69.8%)		
Age groups, years			
<30	48 (12.5%)		
30-39	136 (35.4%)		
40-49	125 (32.6%)		
³ 50	75 (19.5%)		
Residence			
Urban	234 (60.9%)		
Rural	150 (39.1%)		
Education			
Secondary school	65 (16.9%)		
High school	51 (13.3%)		
University	268 (69.8%)		
Employment status			
Yes	219 (57.0%)		
No	165 (43.0%)		
Marital status			
Married	264 (68.8%)		
Single	91 (23.7%)		
Widow/widower	29 (7.6%)		
Children			
0	134 (34.9%)		
1	98 (25.5%)		
2	129 (33.6%)		
3	19 (4.9%)		
4	4 (1.0%)		
Type of disease			
Primary	31 (8.1%)		
Secondary	13 (3.4%)		
RRMS	337 (87.8%)		
Other	3 (0.8%)		
MS duration groups, years			
<5	172 (44.8%)		
5-9	112 (29.2%)		
³ 10	100 (26.0%)		
Treatment groups			
Yes	143 (37.2%)		
No	241 (62.8%)		
Status of a person with a disability			
Yes	31(8.1%)		
No	353 (91.9%)		
	Means ± SD	Median	Min-Max
Age, years	40.3 ± 9.1	40.0	20-67
Age at MS diagnosis, years	33.7 ± 8.5	33.0	20-60
MS duration, years	6.6 ± 5.3	5.0	0-28
EDSS score	2.8 ± 1.4	2.5	0-8

Completing the questionnaire took an average of 12.5 ± 5.4 minutes. Overall, 78.6% of the entire patient population completed the MSQOL-54-GEO questionnaire without assistance. Individuals who suffered with visual perception and cognitive dysfunction needed support.

Table 2 shows the average scores for the MSQOL-54-GEO domains and composite subscales (physical and mental health), alongside the corresponding Cronbach's α coefficients. The "Health Perceptions" component had the lowest average value (47.3 ± 20.0), whereas the "Sexual Function" component had the highest average value of 75.9 ± 28.4. The mean composite score for "Physical Health" was 62.8 with a standard deviation of 21.8, and the mean composite score for "Mental Health" was 64.0 with a standard deviation of 23.0. In general, the Cronbach α values for all MSQOL-54-GEO components are above 0.70, with the exception of the "Social Function" component, which had a Cronbach's α value of 0.645, indicating insufficient internal consistency. The variables "Physical Health" (α = 0.972), "Role Limitations Due to Physical Problems" (α = 0.913), "Health Distress" (α = 0.955), and "Sexual Function" (α = 0.948) all demonstrated excellent internal consistency.

**Table 2:** Mean values of MSQOL-54-GEO subscales and their internal consistency (n = 384) MSQOL-54-GEO: Georgian version of the Multiple Sclerosis Quality of Life-54 questionnaire

#	Subscale	Mean ± SD	Cronbach's a	95% CI for Cronbach's α		Internal consistency
1	Physical health	69.2 ± 32.7	0.972	0.967	0.976	Excellent
2	Role limitations due to physical problems	59.8 ± 43.6	0.913	0.897	0.926	Excellent
3	Role limitations due to emotional problems	64.1 ± 43.1	0.880	0.857	0.899	Good
4	Pain	74.6 ± 25.8	0.837	0.806	0.863	Good
5	Emotional well-being	60.4 ± 18.4	0.792	0.756	0.822	Acceptable
6	Energy	52.3 ± 18.7	0.758	0.717	0.793	Acceptable
7	Health perceptions	47.3 ± 20.0	0.730	0.684	0.770	Acceptable
8	Social function	69.1 ± 22.9	0.645	0.577	0.701	Questionable
9	Cognitive function	75.5 ± 21.7	0.896	0.877	0.912	Good
10	Health distress	63.9 ± 29.1	0.955	0.947	0.961	Excellent
11	Sexual function	75.9 ± 28.4	0.948	0.939	0.956	Excellent
12	Overall quality of life	60.4 ± 23.5	0.870	0.842	0.894	Good
	Composites					
13	Physical health composite	62.8 ± 21.8	0.884	0.865	0.900	Good
14	Mental health composite	64.0 ± 23.0	0.825	0.795	0.850	Good

Table 3 shows the internal validity analysis, using Pearson's R correlation coefficients, which revealed that all components displayed item internal consistency, with R values exceeding 0.100 (ranged 0.66-0.96). The assessment revealed that the "Sexual Function" (D11) domain had lower R values compared to the other domains and subscales, indicating a lower level of discriminant validity. Cultural and social characteristics, for example, could have an impact on the result. It may be regarded as a subject for further research. The correlation analysis indicated that the variables "Role Limitations Due to Physical Problems" (D2) from the Composite subscale "Physical Health" (CS1) and "Role Limitations Due to Emotional Problems" (D3) from the Composite subscale "Mental Health" (CS2) had more positive relationships with other domains and subscales.

**Table 3:** Internal validity assessment (Pearson’s R correlation coefficients) between the MSQOL-54-GEO domains and subscales (n = 384) MSQOL-54-GEO: Georgian version of the Multiple Sclerosis Quality of Life-54 questionnaire

	Subscale	D1	D2	D3	D4	D5	D6	D7	D8	D9	D10	D11	D12	CS1	CS2
	Physical health		0.692	0.541	0.572	0.449	0.573	0.524	0.598	0.423	0.598	0.223	0.666	0.843	0.635
	Role limitations due to physical problems	0.692		0.790	0.570	0.503	0.629	0.566	0.625	0.467	0.625	0.324	0.698	0.865	0.776
	Role limitations due to emotional problems	0.541	0.790		0.556	0.614	0.645	0.601	0.666	0.509	0.666	0.420	0.638	0.791	0.898
	Pain	0.572	0.570	0.556		0.359	0.479	0.429	0.567	0.525	0.567	0.278	0.589	0.733	0.615
	Emotional well-being	0.449	0.503	0.614	0.359		0.724	0.489	0.690	0.610	0.690	0.368	0.552	0.644	0.817
	Energy	0.573	0.629	0.645	0.479	0.724		0.645	0.635	0.443	0.635	0.349	0.572	0.771	0.738
	Health perceptions	0.524	0.566	0.601	0.429	0.489	0.645		0.719	0.454	0.719	0.434	0.666	0.764	0.697
	Social function	0.598	0.625	0.666	0.567	0.690	0.635	0.719		0.575	0.652	0.295	0.716	0.841	0.763
	Cognitive function	0.423	0.467	0.509	0.525	0.610	0.443	0.454	0.575		0.720	0.257	0.586	0.612	0.747
	Health distress	0.598	0.625	0.666	0.567	0.690	0.635	0.719	0.652	0.720		0.420	0.705	0.831	0.868
	Sexual function	0.223	0.324	0.420	0.278	0.368	0.349	0.434	0.295	0.257	0.420		0.426	0.479	0.463
	Overall quality of life	0.666	0.698	0.638	0.589	0.552	0.572	0.666	0.716	0.586	0.705	0.426		0.819	0.806
	Physical health composite	0.843	0.865	0.791	0.733	0.644	0.771	0.764	0.841	0.612	0.831	0.479	0.819		0.889
	Mental health composite	0.635	0.776	0.898	0.615	0.817	0.738	0.697	0.763	0.747	0.868	0.463	0.806	0.889	
	Subscale	D1	D2	D3	D4	D5	D6	D7	D8	D9	D10	D11	D12	CS1	CS2
	Physical health		0.692	0.541	0.572	0.449	0.573	0.524	0.598	0.423	0.598	0.223	0.666	0.843	0.635
	Role limitations due to physical problems	0.692		0.790	0.570	0.503	0.629	0.566	0.625	0.467	0.625	0.324	0.698	0.865	0.776
	Role limitations due to emotional problems	0.541	0.790		0.556	0.614	0.645	0.601	0.666	0.509	0.666	0.420	0.638	0.791	0.898
	Pain	0.572	0.570	0.556		0.359	0.479	0.429	0.567	0.525	0.567	0.278	0.589	0.733	0.615
	Emotional well-being	0.449	0.503	0.614	0.359		0.724	0.489	0.690	0.610	0.690	0.368	0.552	0.644	0.817
	Energy	0.573	0.629	0.645	0.479	0.724		0.645	0.635	0.443	0.635	0.349	0.572	0.771	0.738
	Health perceptions	0.524	0.566	0.601	0.429	0.489	0.645		0.719	0.454	0.719	0.434	0.666	0.764	0.697
	Social function	0.598	0.625	0.666	0.567	0.690	0.635	0.719		0.575	0.652	0.295	0.716	0.841	0.763
	Cognitive function	0.423	0.467	0.509	0.525	0.610	0.443	0.454	0.575		0.720	0.257	0.586	0.612	0.747
	Health distress	0.598	0.625	0.666	0.567	0.690	0.635	0.719	0.652	0.720		0.420	0.705	0.831	0.868
	Sexual function	0.223	0.324	0.420	0.278	0.368	0.349	0.434	0.295	0.257	0.420		0.426	0.479	0.463
	Overall quality of life	0.666	0.698	0.638	0.589	0.552	0.572	0.666	0.716	0.586	0.705	0.426		0.819	0.806
	Physical health composite	0.843	0.865	0.791	0.733	0.644	0.771	0.764	0.841	0.612	0.831	0.479	0.819		0.889
	Mental health composite	0.635	0.776	0.898	0.615	0.817	0.738	0.697	0.763	0.747	0.868	0.463	0.806	0.889	

Table 4 demonstrates the correlation analysis and statistically significant connections between the EDSS score and MSQOL-54 GEO questionnaire components, including "Physical Health" (D1), "Role Limitation Due to Physical Problems" (D2), "Pain" (D3), "Social Function" (D8), "Overall Quality of Life" (D12), and the composite score for "Physical Health" (CS1). The association between the EDSS score and "Sexual Function" (D11) was nearly significant, with a correlation coefficient of -0.095 and a p-value of 0.063. The correlation for this association was consistently negative in all statistically significant instances.

**Table 4:** External validity assessment (Pearson’s R correlation coefficients) between MSQOL-54-GEO domains and subscales (n = 384) vs. EDSS score MSQOL-54-GEO: Georgian version of the Multiple Sclerosis Quality of Life-54 questionnaire, EDSS: Expanded Disability Status Scale

#	Subscale	EDSS	
		R	P
D1	Physical health	-0.354	<0.001
D2	Role limitations due to physical problems	-0.223	<0.001
D3	Role limitations due to emotional problems	-0.050	0.329
D4	Pain	-0.274	<0.001
D5	Emotional well-being	0.042	0.412
D6	Energy	-0.062	0.226
D7	Health perceptions	-0.073	0.153
D8	Social function	-0.265	<0.001
D9	Cognitive function	0.007	0.891
D10	Health distress	-0.041	0.423
D11	Sexual function	-0.095	0.063
D12	Overall quality of life	-0.220	<0.001
CS1	Physical health composite	-0.249	<0.001
CS2	Mental health composite	0.060	0.241

Table 5 presents the results of the external validity assessment between the MSQOL-54-GEO domains and subscales.

Correlation analysis showed (Table 5) statistically significant results between age and “Physical Health” (D1), “Role Limitation Due to Physical Problems” (D2), “Social Function” (D8), and composite score “Physical Health” (CS1); disease duration and “Physical Health” (D1), “Role Limitation Due to Physical Problems” (D2), and “Cognitive Function” (D9); and employment and “Physical Health” (D1), “Pain” (D4), “Social Function” (D8), “Overall Quality of Life” (D12), and composite score “Physical Health” (CS1).

**Table 5:** External validity assessment (Pearson’s R correlation coefficients) between MSQOL-54-GEO domains and subscales (n = 384) MSQOL-54-GEO: Georgian version of the Multiple Sclerosis Quality of Life-54 questionnaire

#	Subscale	Age		Duration of disease		Occupation	
		R	R	R	p	R	P
D1	Physical health	-0.220	-0.211	-0.211	<0.001	0.167	0.001
D2	Role limitations due to physical problems	-0.119	-0.137	-0.137	0.007	0.085	0.096
D3	Role limitations due to emotional problems	0.058	-0.038	-0.038	0.458	0.029	0.571
D4	Pain	-0.069	-0.012	-0.012	0.815	0.166	0.001
D5	Emotional well-being	0.020	0.079	0.079	0.122	-0.089	0.081
D6	Energy	-0.011	0.058	0.058	0.257	0.010	0.845
D7	Health perceptions	-0.028	-0.035	-0.035	0.494	0.017	0.740
D8	Social function	-0.129	-0.096	-0.096	0.060	0.121	0.018
D9	Cognitive function	0.024	0.117	0.117	0.022	-0.023	0.653
D10	Health distress	-0.040	0.011	0.011	0.830	0.047	0.358
D11	Sexual function	0.014	0.046	0.046	0.369	0.064	0.211
D12	Overall quality of life	-0.070	-0.036	-0.036	0.482	0.114	0.026
CS1	Physical health composite	-0.121	-0.095	-0.095	0.063	0.118	0.021
CS2	Mental health composite	0.014	0.013	0.013	0.800	0.018	0.725

Table 6 shows the comparison of internal consistency of MSQOL-54 and Vickrey as baseline data. All the domains are comparable, except social function. The association between disease duration and the composite scores for "Social Function" (D8) and "Physical Health" (CS1) was nearly significant enough to reject the null hypothesis (r = -0.096, p = 0.060; r = -0.095, p = 0.063, respectively).

**Table 6:** Comparison of the internal consistency of MSQOL-54-GEO and Vickrey et al. subscales (n = 384) Citation: Vickrey BG, Hays RD, Harooni R, Myers LW, Ellison GW. A health-related quality of life measure for multiple sclerosis. Qual Life Res. 1995;4:187-206. 10.1007/BF02260859 MSQOL-54-GEO: Georgian version of the Multiple Sclerosis Quality of Life-54 questionnaire

#	MSQOL-54 subscale	Cronbach's a	
		Our study	Vickrey et al. [5]
1	Physical health	0.972	0.96
2	Role limitations due to physical problems	0.913	0.86
3	Role limitations due to emotional problems	0.880	0.84
4	Pain	0.837	0.92
5	Emotional well-being	0.792	0.87
6	Energy	0.758	0.84
7	Health perceptions	0.730	0.80
8	Social function	0.645	0.75
9	Cognitive function	0.896	0.90
10	Health distress	0.955	0.91
11	Sexual function	0.948	0.85
12	Overall quality of life	0.870	0.86
	Composites		
13	Physical health composite	0.884	*
14	Mental health composite	0.825	*

Table 7 presents the results of the physical health MSQOL-54-GEO domains and subscales by gender, showing significant gender differences across most subscales, including Physical Function, Health Perceptions, Energy, Pain, Sexual Function, Social Function, Health Distress, and Total Physical Health Composite Score. Male participants scored higher on average in these domains compared to female participants, with statistically significant differences (p < 0.05) for all except "Role Limitations Due to Physical Problems."

**Table 7:** Results of the physical health MSQOL-54-GEO domains and subscales (n = 384) by gender MSQOL-54-GEO: Georgian version of the Multiple Sclerosis Quality of Life-54 questionnaire

Subscale	Females		Males		T	P
	Mean	SD	Mean	SD		
Physical function	66.1	35.1	76.7	28.9	2.87	0.004*
Health perceptions	45.1	19.9	52.4	19.5	3.33	0.001*
Energy	50.5	17.8	56.5	20.0	2.93	0.004*
Role limitations due to physical problems	56.5	43.3	67.9	43.5	1.25	0.210
Pain	72.0	25.8	80.9	24.7	2.79	0.006*
Sexual function	72.1	29.9	84.7	22.2	4.09	<0.001*
Social function	67.4	22.9	72.9	22.7	2.17	0.031*
Health distress	61.3	29.2	70.1	28.2	2.75	0.006*
Total physical health composite score	60.3	21.6	69.0	21.2	3.65	<0.001*

Table 8 presents the results of the mental health MSQOL-54-GEO domains and subscales by gender. Although male participants also scored higher on several mental health subscales, significant gender differences were observed only in "Health Distress" and "Overall Quality of Life," where males had higher mean scores than females. Differences in "Emotional Well-being," "Role Limitations Due to Emotional Problems," and "Cognitive Function" were not statistically significant. 

**Table 8:** Results of the Mental Health MSQOL-54-GEO domains and subscales (n = 384) by gender MSQOL-54-GEO: Georgian version of the Multiple Sclerosis Quality of Life-54 questionnaire

Subscale	Females		Males		t	p
	Mean	SD	Mean	SD		
Health distress	61.3	29.2	70.1	28.2	2.75	0.006*
Overall quality of life	57.4	23.0	66.4	23.6	3.50	<0.001*
Emotional well-being	60.1	18.1	61.5	19.3	0.68	0.495
Role limitations due to emotional problems	64.4	42.5	63.8	44.5	0.13	0.900
Cognitive function	74.8	23.1	77.3	18.4	1.04	0.301
Total mental health composite score	63.0	22.5	66.5	24.1	1.37	0.171*

## Discussion

The Cronbach's α values in the study are nearly comparable, with the exception of the "Social Function" category. The limited social activity possibilities available to MS patients in Georgian society may explain the low Cronbach's score for the social function component. The low Cronbach's α value for the "Social Function" domain could be attributed to the limited number of items (three) that measure the area. Furthermore, the structure of these questions, which covered broader and less specific topics, resulted in a significant loss in overall coherence.

In comparison to the female group, the male group performed significantly better on all subscales of the physical health component of MSQOL-54-GEO, including the "Total Physical Health Composite Score." The only exception was the lack of statistical significance in the disparity in scores for "Role Limitations Due to Physical Problems" (p > 0.05).

In comparison to the female group, the male group scored considerably higher on the MSQOL-54-GEO's mental health component subscales of health distress, overall quality of life, and total mental health composite score. There was no statistically significant difference in the scores for "Emotional Well-being," "Role Limitations Due to Emotional Problems," and "Cognitive Function" (p > 0.05).

The majority of respondents were able to complete the questionnaire within the recommended time frame of 11-18 minutes [5,9-12], indicating that the questions and their translated counterparts were comprehensible and feasible to complete. The involvement and support of clinicians throughout the process contributed to a decrease in incomplete data. Solari et al. [6] performed a validation study in Italy [12], whereas Idiman et al. [11] conducted a study in Turkey. Pekmezovic T et al. [8,13] conducted a study in Serbia. Catic et al. [9,14] conducted a study in Slovenia [10,15]. Catic T et al. did research in Bosnia and Herzegovina, whereas Estiasar et al. [16] conducted a study in Indonesia [17]. Each of these studies indicated low internal consistency for the "Sexual Function" measure. In their surveys, the questions on this topic and the level of satisfaction with sexual performance were most likely to be missing data. Furthermore, they stated that this issue originated as a result of the shame associated with acknowledging sexual matters, aligned to current societal standards. To tackle this issue in our study, trained physicians provided instruction and support to the study participants while collecting answers to specific questions (questions 20, 33, and 51). 

The study findings reveal significant insights into the QOL of patients with MS in Georgia, which can be compared to studies conducted in other countries. Similar to findings in studies from the United States and European countries, such as Slovenia and Bosnia and Herzegovina, research shows that physical limitations and emotional distress significantly affect the QOL in MS patients. However, one unique aspect of the study is the cultural context of Georgia, where familial support plays a critical role in coping with the disease, as highlighted in the findings. These cultural nuances are less prominent in studies from Western countries, where patients are more likely to rely on formal healthcare systems. There was additionally a stronger association between depression and reduced QOL among Georgian patients compared to findings in studies from Scandinavian countries, where healthcare support systems may offer more extensive psychiatric services. The inclusion of a culturally adapted version of the MSQOL-54 also provides new insights into the importance of context-specific modifications for the accurate assessment of HRQOL. 

The study found a negative correlation between the EDSS and the composite scores of "Physical Health." Based on the data and study results, it could be reasonable to conclude that the EDSS score can be utilized to predict physical health difficulties. Higher EDSS scores correlate with lower composite scores for "Physical Health." An analysis of age, disease duration, and occupation revealed a strong correlation with multiple components of "Physical Health," such as "Physical Health," "Role Limitations Due to Physical Problems," "Social Function," and the overall composite score of "Physical Health." Regarding the highlighted internal consistency concerns of "Social Function," the correlation analysis revealed a favorable link between occupation and "Social Function." Employed persons had higher values in this domain compared to housewives and non-employed adults with MS.

Study limitations

Due to the centralized healthcare system delivery, MS patients were enrolled during the visits in the urban areas, and the results may not directly show the exact picture of the social burden of the whole country. Patients with high EDSS 8-10 were not included due to the inability to visit a doctor, and the study lacked demographic information about economic income, ethnicity, and treatment approach.

Strength of the study

The study presents several strengths that enhance its value in understanding the QOL of MS patients in Georgia. First, the validation of the Georgian version of the MSQOL-54 is a significant strength. This culturally adapted instrument provides a reliable and valid measure of HRQOL in the Georgian population, which is crucial for both clinical practice and research in the country. Second, the study’s large sample size and diverse participant demographics ensure that our findings are robust and representative of the broader MS patient population in Georgia. By including both urban and rural patients, we were able to capture the varying healthcare access and sociocultural differences that influence HRQOL.

Another strength of the study is its interdisciplinary approach, with the potential of exploration of multiple dimensions of MS-related QOL, offering a comprehensive view of the patient experience. In addition, the focus on cultural adaptation in the validation process ensures that the MSQOL-54 is not only linguistically appropriate but also contextually relevant for Georgian patients. This is a significant contribution to the global body of MS research, where few studies have specifically addressed the cultural nuances in HRQOL assessments.

## Conclusions

The MSQOL-54-GEO is a reliable tool for assessing QOL in MS patients in Georgia, the results demonstrate that the MSQOL-54-GEO has a high level of dependability, as proven by its internal consistency.

This is the first study to holistically evaluate the QOL assessment tool for MS patients in Georgia. The MSQOL-54-GEO has demonstrated strong validity and reliability in assessing the HRQOL of MS patients in Georgia. In addition, this questionnaire serves as a valuable initial reference for physicians, addressing not only clinical aspects but also medical rehabilitation, cognitive function, and psychological concerns. The study provides significant findings regarding the internal consistency of the MSQOL-54-GEO questionnaire, particularly in regard to physical health, emotional well-being, health perceptions, and social function. A study assessed the HRQOL and social stress encountered by individuals with MS in Georgia. The results unveiled complex interconnections between disability, QOL, and sociodemographic determinants. The study highlights the imperative of customized interventions, support networks, and public health initiatives to mitigate the total social burden of MS. Collaboration between healthcare experts, legislators, and the public sector can improve the overall well-being and QOL of those affected by MS by acknowledging and tackling the various issues they face.

By implementing a validated local (Georgian) version of the MSQOL-54, the healthcare system can provide a more patient-centered approach, improving treatment outcomes and overall well-being for MS patients.

The use of the MSQOL-54 in this study provides a unique opportunity to enhance patient-centered care by offering a more comprehensive understanding of the QOL of MS patients in Georgia. Healthcare providers can integrate this tool to assess individual patient needs, improve treatment planning, and prioritize holistic care, such as psychological support and physical therapy, thus promoting better overall well-being and treatment outcomes.
